# ‘Distraction Vaginogenesis’: Preliminary Results Using a Novel Method for Vaginal Canal Expansion in Rats

**DOI:** 10.3390/bioengineering10030351

**Published:** 2023-03-12

**Authors:** Hannah Meyer, Lexus Trosclair, Sean D. Clayton, Collyn O’Quin, Zachary Connelly, Ross Rieger, Nhi Dao, Ahmed Alhaque, Andrew Minagar, Luke A. White, Giovanni Solitro, Mila Shah-Bruce, Valerie L. Welch, Stephanie Villalba, Jonathan Steven Alexander, Donald Sorrells

**Affiliations:** 1 Department of Surgery, LSU Health Shreveport, Shreveport, LA 71103, USA; 2 Department of Molecular and Cellular Physiology, LSU Health Shreveport, Shreveport, LA 71103, USA; 3 Department of Orthopedic Surgery, LSU Health Shreveport, Shreveport, LA 71103, USA; 4 Department of Obstetrics and Gynecology, LSU Health Shreveport, Shreveport, LA 71103, USA; 5 Department of Pathology, LSU Health Shreveport, Shreveport, LA 71103, USA; 6 Department of Biology, Louisiana State University in Shreveport, Shreveport, LA 71115, USA

**Keywords:** vaginal agenesis, vaginal atresia, vaginal dilation, vaginal elongation, Mayer–Rokitansky–Küster–Hauser syndrome, androgen insensitivity syndrome, tissue reconstruction

## Abstract

Vaginal atresia is seen in genetic disorders such as Mayer–Rokitansky–Küster–Hauser (MRKH) syndrome, which can cause significant sexual dysfunction. Current treatments include surgical reconstruction or mechanical dilation of the vaginal canal. Mechanical dilation requires patients to be highly motivated and compliant while surgical reconstruction has high rates of complications. This study evaluated a novel vaginal expansion sleeve (VES) method as an alternative treatment for vaginal atresia. The proprietary cylindrical VES is a spring-like device consisting of polyethylene terephthalate helicoid trusses capped at each end with a fixed diameter resin cap for fixation within tissues. Following the development of the VES and mechanical characterization of the force–length relationships within the device, we deployed the VES in Sprague Dawley rat vaginas anchored with nonabsorbable sutures. We measured the VES length–tension relationships and post-implant vaginal canal expansion ex vivo. Vaginal histology was examined before and after implantation of the VES devices. Testing of 30 mm sleeves without caps resulted in an expansion force of 11.7 ± 3.4 N and 2.0 ± 0.1 N at 50% and 40%, respectively. The implanted 20 mm VES resulted in 5.36 mm ± 1.18 expansion of the vaginal canal, a 32.5 ± 23.6% increase (*p* = 0.004, Student *t* test). Histological evaluation of the VES implanted tissue showed a significant thinning of the vaginal wall when the VES was implanted. The novel VES device resulted in a significant expansion of the vaginal canal ex vivo. The VES device represents a unique alternative to traditional mechanical dilation therapy in the treatment of vaginal atresia and represents a useful platform for the mechanical distension of hollow compartments, which avoids reconstructive surgeries and progressive dilator approaches.

## 1. Introduction

Vaginal atresia is a congenital absence or underdevelopment of the vaginal canal and is commonly seen in genetic disorders such as Mayer–Rokitansky–Küster–Hauser (MRKH) syndrome or complete androgen insensitivity syndrome (CAIS) [1,2]. MRKH syndrome is a rare congenital disorder that occurs in approximately one in 4000–5000 female births. Females with MRKH have vaginal aplasia and other Mullerian (i.e., paramesonephric) duct abnormalities. Women with MRKH typically lack a fully uterus and vaginal canal, but are otherwise externally phenotypically and genotypically normal with all other secondary sex characteristics [3,4]. CAIS is another congenital malformation that occurs in about one in every 20,000 male births. CAIS is characterized by undescended testes and phenotypically female external genitalia in genotypic males. In CAIS, the vaginal canal is shorter (less than 8 cm) than that in normal women and is blind ending [5,6]. Partial androgen insensitivity syndrome (PAIS) is also characterized by atypical genitalia, which may also require reconstruction based on the gender identity of the patient. The absence of a vaginal canal in MRKH, CAIS, and PAIS can result in severe sexual dysfunction in adolescence and adulthood with profound psychosocial stresses [3]. Currently, available treatments for such forms of vaginal atresia include reconstructive surgical and non-surgical options.

The current first-line treatment for vaginal agenesis is serial mechanical dilation of the vaginal canal where pressure and hard dilators are inserted incrementally into the vagina to progressively increase both the length and diameter of the vaginal canal [7]. Frank first described dilation therapy in 1938, which has since become more widely used [8]. Procedural success, determined as a functional vaginal canal, does not depend on the starting vaginal length, but depends on significant patient compliance and support to perform 30-min of dilation up to three times a day [2]. Treatment can take an average of about six months when compliant, with one study of 245 patients describing a range of two to nineteen months [4]. Because of this need for patient cooperation, previous literature has suggested that therapy begins after the patient is evaluated by a psychologist for emotional maturity and is determined to have adequate intrinsic motivation to avoid a psychological barrier to dilator treatment or to avoid exacerbation of pre-existing psychological hardships as a result of their diagnosis [4,9]. Primary dilation is considered first-line due to lower morbidity and costs than surgical therapy [2,7,10,11]. Common complications are less severe than those of surgery and consist of urethral irritation and urinary leakage [2,4]. Despite being safer and more cost-effective, dilation is not universally successful, and some cases require a surgical approach.

Various techniques for surgical vaginoplasty exist. The type of procedure a patient undergoes depends on several factors including surgeon preference, the patient’s clinical condition, and the patient’s age [12]. A previous study of 131 patients found that surgical vaginoplasty had a 40% higher rate of complications compared to primary dilation therapy [13]. Many of these complications will subsequently require secondary operations for correction [2]. One laparoscopic method is the Vecchietti procedure, which utilizes a small olive-shaped bead attached to threads connected to a traction device on the abdominal wall that are then tightened about 1 cm per day for 7–10 days or until a satisfactory length is reached (7–10 cm) [1,14,15]. Due to the pain associated with continuous traction, the patient must stay in the hospital throughout the traction process [15]. Moreover, potential significant complications such as bladder and rectal lesions are possible due to the limited retrovesicorectal space where the threads are placed [14,15].

The Abbe–McIndoe procedure, an open method, consists of dissecting out the potential space of the neovagina followed by a skin graft to create the new vaginal wall. This procedure may be accompanied by serious complications such as infection from the contact between the vagina and peritoneal cavity, infection of the graft, and neovaginal stenosis [14,15,16]. A more complicated and invasive method is intestinal vaginoplasty, which uses a segment of the bowel, usually the sigmoid, ileum, or jejunum, to create a neovagina [14]. Serious complications of the intestinal vaginoplasty include intra-abdominal hemorrhage, intestinal obstructions, and prolapse of the vaginoplasty architecture [14,15,16]. One major advantage that sets the intestinal vaginoplasty apart from the Vecchietti and Abbe–McIndoe procedures is the lack of possible vaginal contraction post-operatively. Therefore, the patient is not required to complete manual dilation after surgery to maintain vaginal patency [14,15]. Generally, other forms of vaginoplasty require mechanical dilation therapy after surgery to maintain vaginal length and patency, and post-surgical dilation compliance is of the utmost importance to avoid future surgical procedures and complications [7].

Successful treatment has previously been classified into two categories: anatomical success and functional success. Anatomical success is largely defined as a vaginal canal of adequate length, typically at least 7 cm [17]. Functional success is considered more meaningful and is defined as the ability to achieve successful and satisfactory intercourse or the ability to accept the largest dilator without pain or discomfort [17,18,19,20]. Studies have found no correlation between the initial vaginal length and success of mechanical dilation, indicating that a vaginal dimple or less is sufficient with a motivated patient [18,20]. The frequency of dilation treatments seems to have a high influence, with one study reporting a greater change in vaginal length, a higher functional success rate, and a shorter duration of treatment for those patients that routinely completed their dilation therapy [17]. Although primary dilation has proven to be successful, surgery has been found to have a higher rate of functional success than dilation therapy [19].

Because of the potential surgical complications and difficulty of patient compliance, we proposed the use of a modification of our previously described intestinal expansion sleeve method [21] to expand the vaginal canal length in a single step procedure in rats. We hypothesized that the vaginal expansion sleeve (VES) would induce lengthening of the vaginal canal without lesion to the vaginal wall through the process of ‘distraction vaginogenesis’, a derivative of distraction enterogenesis described in studies focused on treating short gut syndrome with longitudinal expansion devices that lengthen the bowel through distractive mechanical forces [21,22]. In the attempt to evaluate a novel self-expanding device as a potential treatment option for vaginal agenesis, this proof-of-concept study aims to create and mechanically characterize the VES device and to histologically evaluate the expansion of the vaginal wall following deployment.

## 2. Materials and Methods

**Figure 1:**
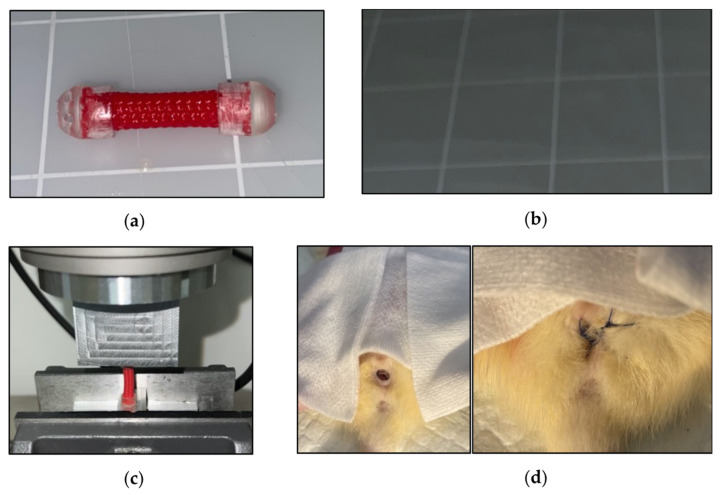
(**a**) The original VES design inserted into deceased rats. (**b**) Redesigned VES with flex seal coating and one cap. (**c**) Biomechanical characterization testing of original VES design. (**d**) VES post-insertion into the rat vaginal canal (**left**) and after suturing into place (**right**).

The VES is made of a woven 5 mm cylindrical layered polyethylene terephthalate with helicoid trusses characterized by isometric ends. Initially, each VES was cut to 20 mm in length. Caps comprised of the Biocompatible Photopolymer Resin Surgical Guide (Formlabs, Somerville, MA, USA) were secured onto each end using epoxy (Figure 1a). However, the two caps interfered with the compression of the sleeve. After initial biomechanical testing, the VES was redesigned to have a coating of liquid rubber on each end to prevent fraying of the material, and a cap was kept only at the distal end (Figure 1b) to aid in suturing the device in place at the introitus. Additionally, barium was mixed into the Flex Seal^®^ (Swift Response Inc.; Weston, FL, USA) to allow for post-operative visualization of the device.

Mechanical characterization of the VES was performed utilizing an Instron 8874 Biaxial Servo Hydraulic Fatigue Testing System. Cyclical compression and decompression of the VES device at a rate of 50 mm/min determined the force load (in newtons) that VES exerted at 10%, 20%, 30%, 40%, and 50% compression (Figure 1c).

VES devices were implanted in the vaginal canal of deceased Sprague Dawley rats. The device was pre-contracted by diametric expansion before being deployed into the vaginal canal. Post-insertion, the VES was anchored and secured using three nonabsorbable 4-0 silk sutures at the exterior end of the vaginal canal (Figure 1d). The vaginal canals were then harvested, post-expansion canal lengths were measured ex vivo, and tissue was prepared for preliminary histological analysis.

Vaginal canal tissues were fixed in 3.7% phosphate buffered formaldehyde for 24 h and then transferred to the LSU Pathology Core laboratory. Specimens were transferred through graded alcohol series and then into paraffin. Sections 5 μm in size were cut and processed, deparaffinized, and then stained for hematoxylin/eosin and mounted on slides. Photographs of specimens were taken, and the tissue layer thicknesses were evaluated using ImageJ (NIH, Bethesda, MD, USA, https://imagej.nih.gov/ij/) (accessed on 24 June 2022). Statistical differences between treatment groups were analyzed using the two-tailed Students’ *t*-test. A *p*-value of 0.05 or less was considered significant.

## 3. Results

### 3.1. Mechanical Characterization

**Figure 2:**
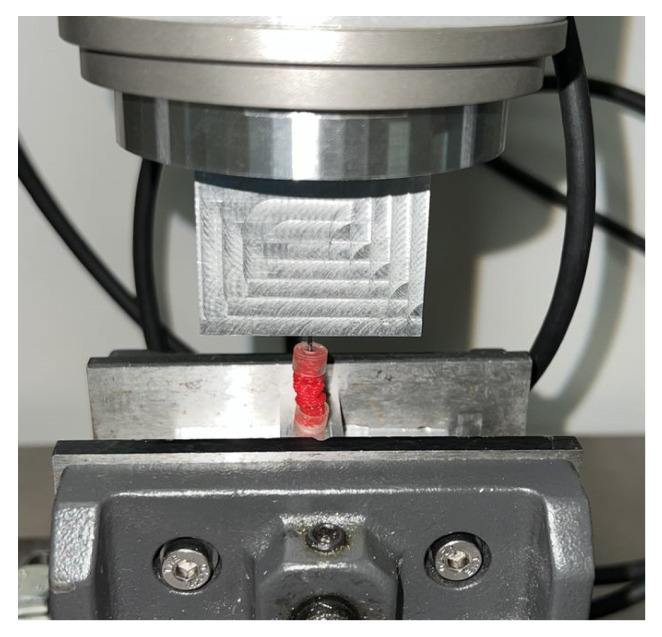
Failed re-expansion of double-capped VES post-compression.

Testing of the biomechanical characterization of VES revealed that the caps on each end of the sleeve not only compromised the sleeve’s compression but also restricted the sleeve from re-expanding to its original length after compression (Figure 2). For this reason, further biomechanical testing was conducted using sleeves without caps on each end and with sleeves cut to 30 mm instead of 20 mm to compensate for the loss in cap length. The length of each device was measured pre- and post-compression with calipers to verify the post-compression expansion to the original length. The average nominal length of each VES was 30.13 mm, and the average post-compression length of each VES was 29.86 mm. The expansive force of each sleeve was recorded at 10%, 20%, 30%, 40%, and 50% compression (Table 1).

### 3.2. Ex Vivo Insertion

**Figure 3:**
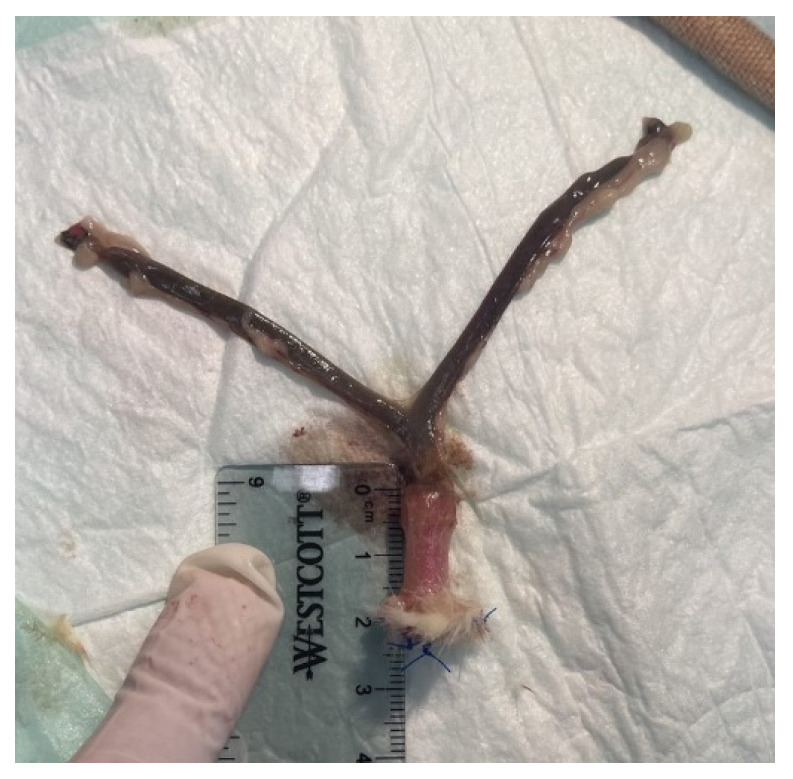
Harvested vaginal canal with the inserted VES.

Seven VES devices were inserted into the vaginal canals of deceased Sprague Dawley rats and then the post-insertion vaginal lengths were immediately measured ex vivo (Figure 3). The mean pre-insertion vaginal length was 18.29 ± 3.35 mm, and the mean post-insertion vaginal length was 23.64 ± 2.43 mm, resulting in a mean vaginal lengthening of 5.36 ± 1.18 mm (Table 2). There was a 32.5 ± 23.6% increase (*p* = 0.004, Students *t*-test) in the vaginal length from pre- to post-insertion.

### 3.3. Histological Analysis

**Figure 4:**
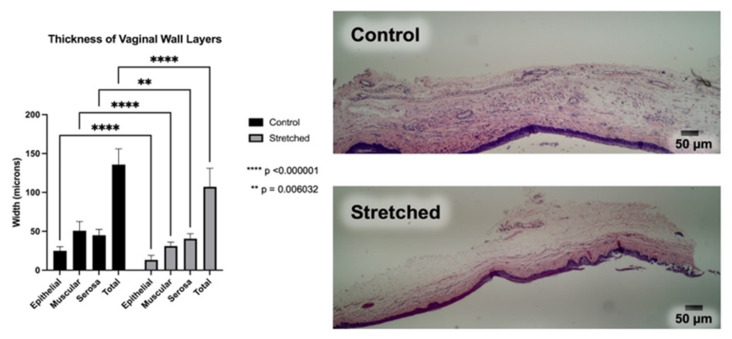
Tissue layer thickness in the control and stretched vaginal tissue, assessed on the hematoxylin and eosin-stained slides.

Histology demonstrated significant thinning across the epithelial, muscular, and serosal layers when compared to the control vaginal tissue samples, resulting in total vaginal wall thinning (Figure 4 and Table 3) in all tissue layers.

**Figure 5:**
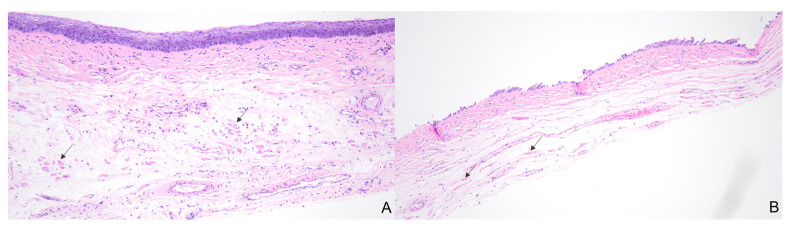
Black arrows show the muscle fibers in the control (**A**) and stretched (**B**) vaginal wall tissue. (**A**) Control tissue with unremarkable overall tissue width, organized, maturing squamous mucosa, and bundles of muscle fibers with some interspersed connective tissue (H&E, ×100). (**B**) Stretched tissue with reduced overall width, squamous disruption with only a partial basal layer remaining intact, and muscle fibers that are elongated and thinner than the control muscle fibers (H&E, ×100).

Review of the hematoxylin and eosin (H&E) stained slides revealed significant thinning of the overall vaginal wall as well as thinning of the individual histologic layers of the vaginal wall tissue following VES insertion. The control tissue showed organized, maturing squamous mucosa with a well-defined basal layer. The submucosa showed loose fibrous connective tissue with a zone of muscle fibers in loosely-formed bundles and blood vessels scattered throughout (Figure 5A). The stretched tissue showed near-complete denudation of the squamous mucosa with an incomplete basal cell layer remaining attached to the underlying soft tissue. The depicted squamous mucosa is most representative of the stretched tissue, but occasional areas in some rats show more residual squamous mucosa, up to full thickness. The submucosa in the stretched tissue is thinner and more compressed than the control tissue, and the muscle fibers are elongated and nearly imperceptible at this magnification (Figure 5B).

## 4. Discussion

Vaginal atresia can lead to significant sexual dysfunction and therefore needs to be treated to attain successful penetrative intercourse. Treatment can lead to improved sexual satisfaction and quality of life [3]. The current treatment options for vaginal atresia have been proven to be successful, but each has its drawbacks. First-line treatment of serial dilation has promising results but relies heavily on patient compliance [2]. Additionally, surgical intervention has higher rates of serious complications and is typically reserved for those who have failed mechanical dilation [7]. In this study, we proposed an alternative therapy to mechanical dilation by utilizing a vaginal expansion sleeve (VES) that mechanically lengthens the vaginal canal in a single step, without relying on patient compliance. The VES may also have an additive role to either dilation therapy or a surgical option. For example, the VES could help to increase vaginal length and diameter in a patient who has not achieved functional vaginal success through other means.

Many patients that present with vaginal atresia have difficulty adjusting to their diagnosis emotionally. Successful creation of a neovagina can relieve a great deal of these psychological stresses, however, patients have reported that the process of dilation therapy serves as a constant reminder that they are ‘abnormal’ [4]. We anticipate that our device can lessen the frequency of this sentiment by performing the dilation therapy without manual effort or continuous attention from the patient. In this way, we hope the VES device can provide an easier path emotionally in the treatment of vaginal atresia. Theoretically, the desired vaginal canal can be achieved by customizing the VES to the patient’s evolving or lengthening vagina. The VES could be 3D printed to exert slowly increasing force to achieve a goal. Multiple VES deployments may be necessary to achieve the optimal canal length. This method of vaginal lengthening could even be used to salvage a patient’s vaginal canal who failed with other therapies.

Our proposed device is derived from another study focused on the use of distraction enterogenesis in the treatment of short gut syndrome through an intestinal expansion sleeve (IES) [21]. Distraction enterogenesis utilizes longitudinal expansion devices that inflict distractive mechanical force for an immediate increase in the small intestine length while maintaining tissue continuity and architecture [22]. We aim to repurpose the use of this IES device for ‘distraction vaginogenesis’ in the treatment of vaginal atresia. Placement of this device in a patient with a limited dimple on the perineum may require initial dilation therapy to develop a ‘landing zone’ for the VES. Thus, the device would be used in a case of severe vaginal atresia after a limited canal has been established to provide a space to deploy.

Testing of the biomechanical characterization revealed that compressing the sleeve to 50% of its original length yielded the highest exertional force load at 11.69 ± 3.45 N. Compressing the VES device to only 40% of its original length resulted in a lower exertional force load at only 1.98 ± 0.11 N, suggesting that the device contraction must be maintained while being inserted and anchored into the vaginal canal in order to achieve maximal lengthening. Anchoring the device to the external vaginal orifice was achieved using non-absorbable sutures to ensure the force exerted was directed inward toward the proximal end of the vagina. The use of absorbable sutures was considered, but once dissolved, the force exerted would be equalized on both ends of the device, causing less force to be directed toward lengthening as the device springs out bidirectionally and the device extrudes out of the vagina. Moreover, biomechanical testing revealed that the post-expansion sleeve length is nearly equivalent to the original pre-compression length. This demonstrates how we can approximate the length of the vaginal canal by adjusting the length of our device. Ex vivo insertion revealed an average of 32.5 ± 23.6% in the immediate increase in vaginal length. However, whether the increased vaginal length is maintained or the vagina returns to its initial length once the device is removed requires further study in a live animal model. Furthermore, live animal trials are warranted to optimize the duration of implantation of the VES device required to elicit a more permanent stretch in the vaginal wall tissue.

Histologic analysis revealed an overall thinning of the vaginal wall tissue following VES insertion. The compression of the soft tissue and elongation of the muscle fibers indicate vaginal stretching and do not show significant connective tissue or muscle damage. However, the squamous mucosa disruption is significant in the stretched vaginal wall tissue. Because the rats in this study were dead at the time of the VES insertion, the vaginal wall tissue was not able to react in a physiological manner. Physical stretching of the vaginal wall tissue is possible, but this study could not address any of the other effects that may occur in the vaginal wall if this study was to be performed in live rats. The significant squamous mucosa changes seen in the stretched tissues in this study may not be seen in live rats, since live rats may exhibit a physiologic counterforce or other protective measures to ensure that the squamous mucosa integrity is maintained during and after the VES insertion. Chemical signaling, inflammatory response, and injury repair would be expected to occur in a live rat, and further studies are necessary to describe these effects. The enduring effects on the vaginal wall and potential permanent remodeling of the vagina following both short- and long-term VES insertion should be examined from a histologic standpoint to determine the optimal use of the VES to benefit patients with the least associated morbidity. There were several limitations of our study. The main limitation is that we used a rat model. The rat vagina typically has a length of around 15–20 mm and a diameter of around 3–5 mm, both of which are smaller than the 6–10 cm length and the 1.5–5 cm diameter typical of a normal human vagina [23]. However, it has been documented that there is an anatomical correspondence that would allow studies in the rat model to be extrapolated to humans [23,24]. In the current study, we measured a maximal force exercised by the VES device of 11.69 ± 3.45 N. This is enough force to stretch the smaller rat vagina, but the force required of a human vagina would be larger, with one study by Cosson et al. finding a human vaginal tensile strength of 44.28 ± 20.29 N [25]. Furthermore, in this study on the murine model, we documented an expansion of 5.36 ± 1.18 mm, which is one order smaller than what has been documented for successful human vaginal lengthening [16]. Although this is a more modest result, reconfiguring the VES to allow greater expansion capability is anticipated, and the length, diameter, and exertional force of the proposed device would therefore need to be scaled to fit human vaginal standards. In light of the scalability needed, the results of our study need to be further corroborated with experiments on human tissues.

Our device requires some depth to the vagina in order to be inserted and may not be deployable in very shallow vaginal dimples. However, we may consider the use of the VES device as an adjunct to mechanical dilation therapy. Studies have found that shallow vaginal dimples are still amenable to traditional dilation therapy, and there is no correlation between the starting vaginal length and the success of dilation [18,20]. Moreover, one study following 16 women through dilation treatment reported that the frequency of dilation therapy significantly decreased to only once a week after 1–3 months of treatment, citing factors such as time and effort constraints, privacy issues, and a lowered perceived vaginal lengthening as reasons to reduce therapy frequency [26]. The reduced frequency in therapy resulted in significantly smaller vaginal lengths than those patients who dilated frequently. Our device could eliminate these negative factors as it does not require the patient to manually perform the treatment. As soon as the patient achieves a minimal length, the VES device can be implanted. Time commitments and issues finding privacy to manually conduct dilation and decreased motivation from a perceived stalled lengthening would essentially be eliminated. The likelihood of a larger end vaginal length would increase, and the need for surgery may be avoided.

Another limitation is that this study was performed on a normal rat vagina and not an atretic one. A vaginal canal with significant atresia will likely be more challenging to elongate. Our initial biomechanical tests were performed on tissue derived from deceased adult rats with fully formed vaginal canals. Hypoplastic vaginal canals may have a different tissue composition of epithelial, smooth muscle, and even substantial scar tissue from prior attempts at treatments that may alter the performance of the VES [12]. Additional effects from VES deployment on vaginal neuronal and vascular supplies would need further study in a live animal model over a longer period of time. Furthermore, we did not obtain full compression of the VES device due to the size of the resin cap. This could be resolved by using an absorbable cap on the proximal end that dissolves after VES anchoring. We will implement this change in future in vivo studies focused on the tissue remodeling over time.

Immediate vaginal elongation was successfully achieved with the VES in deceased Sprague Dawley rats by thinning the vaginal canal tissue without compromising it. VES expansion to its equilibrium dimension maintained longitudinal vaginal canal tension, which may permit remodeling and increased canal length while preserving vascular and nervous supplies. Further studies are needed in vivo to address and verify this theory of ‘distraction vaginogenesis’.

## 5. Conclusions

VES is a unique approach for potentially achieving vaginal canal elongation in women with vaginal atresia and represents a promising alternative or adjunct to primary dilation by eliminating vigilant patient compliance. Devices creating distraction vaginogenesis could even be used to improve a surgically created vaginal canal. The VES device is less invasive than traditional surgical interventions, suggesting that this device represents a viable alternative to mechanical dilation and surgical interventions. However, our results still require further in vivo studies for biocompatibility and mechanical characterization, in order to more fully understand and validate this novel approach.

Future research on the VES will include the determination of the in vivo elongation of the vaginal canal in Sprague Dawley rats. This method of elongation of the vaginal canal may have other patient populations that could benefit such as transgender. The VES could be impregnated with estrogen, VEGF, or GLP-2 to enhance the mucosal stimulation and improve the architecture of the neovagina. Studies will need to be performed to determine if these ‘enhanced’ expansion sleeves have benefits. A VES with dissolvable cross links may be better tolerated in future patients. These cross linked devices would exert force in a slower measured time frame. Further investigation is warranted in order to optimize the duration of implantation and the forced expansion necessary to facilitate ‘distraction vaginogenesis’.

## 6. Patents

The authors (J.S.A., D.S., G.S., L.W., S.V.) have disclosed this technology (“Tissue Expander Sleeve and Treatments for Short Bowel Syndrome, Vaginal Stenosis, Vaginal Agenesis/Atresia and Neovaginal Dilation”) to the LSU Health Office of Sponsored Programs and Technology Transfer.

## Figures and Tables

**Table 1:** Average expansive force of VES at various compression intensities.

Compression (%)	10	20	30	40	50
Force (N)	0.34 ± 0.09	0.74 ± 0.05	1.16 ± 0.09	1.98 ± 0.11	11.69 ± 3.45

**Table 2:** Vaginal canal lengths before VES insertion and after VES insertion.

	1	2	3	4	5	6	7
Pre-insertion (mm)	13	16	16	22	21	21	19
Post-insertion (mm)	21.5	22	26	25	25	26	20
Expansion (%)	65.4	37.5	62.5	13.6	19.0	23.8	5.3

**Table 3:** Vaginal tissue layer widths in the control and stretched tissues.

Tissue Layer	Mean Control (μm)	Mean Stretched (μm)
Epithelial	24.93 ± 5.30	13.17 ± 5.89
Muscular	50.70 ± 11.88	31.01 ± 5.07
Serosa	45.00 ± 7.45	40.29 ± 6.73
Total	135.76 ± 20.23	107.11 ± 23.76

## Data Availability

Not applicable.
